# Synchronous vascular endothelial growth factor protein profiles in both tissue and serum identify metastasis and poor survival in colorectal cancer

**DOI:** 10.1038/s41598-019-40862-6

**Published:** 2019-03-12

**Authors:** Chien-Chih Yeh, Li-Jane Shih, Junn-Liang Chang, Yi-Wei Tsuei, Chang-Chieh Wu, Cheng-Wen Hsiao, Chih-Pin Chuu, Yung-Hsi Kao

**Affiliations:** 1 Department of Life Sciences, National Central University, Taoyuan, 320 Taiwan; 2 Division of Colon and Rectal Surgery, Department of Surgery, Taoyuan Armed Forces General Hospital, Taoyuan, 325 Taiwan; 3 National Defense Medical Center, Tri-Service General Hospital, Taipei, 114 Taiwan; 4 Medical Laboratory, Taoyuan Armed Forces General Hospital, Taoyuan, 325 Taiwan; 5 Department of Pathology and Laboratory Medicine, Taoyuan Armed Forces General Hospital, Taoyuan, 325 Taiwan; 6 Department of Emergency, Taoyuan Armed Forces General Hospital, Taoyuan, 325 Taiwan; 7 Division of Colon and Rectal Surgery, Department of Surgery, Tri-Service General Hospital, Taipei, 114 Taiwan; 8 Institute of Cellular and System Medicine, National Health Research Institutes, Miaoli, 350 Taiwan

## Abstract

Colorectal cancer (CRC) is the third leading cause of cancer-related death worldwide. We examined if tumor tissue and circulating protein levels of all vascular endothelial growth factors (VEGFs) and VEGF receptors (VEGFRs) were synchronous and different in Taiwan patients with metastatic CRC (mCRC) vs. non-mCRC. We analyzed samples from 109 patients enrolled from 2005–2017, 50 with stages I/II and 59 with stages III/IV CRC. We found that VEGF-A, -B, -C, -D, placental growth factor (PlGF), VEGFR-1, VEGFR-2, and VEGFR-3 were higher in tumor tissues than non-tumor tissues. Metastatic patients had higher levels of circulating VEGFs and soluble VEGFRs (sVEGFRs) than healthy subjects, as well as higher VEGF-A, -B, -C, -D, and PlGF proteins in both tumor tissue and serum than non-metastatic patients. Protein levels of VEGF and VEGFR were mainly associated with the patient’s age, tumor site, tumor size, tumor stage, and lymph node metastasis. Patients exhibiting high levels of VEGF, VEGFR, and sVEGFR had a shorter overall survival and disease-free survival than those with low levels. We conclude that synchronous changes in VEGF and VEGFR levels in CRC tissue and serum VEGF can discriminate between metastatic and non-metastatic subjects and high levels are associated with poor survival in CRC.

## Introduction

Colorectal cancer (CRC) is the third leading cause of cancer-related death worldwide, including in Taiwan^1–3^. According to cancer statistics by the World Health Organization (WHO), about 774,000 people died from CRC in 2015^2^; while, the WHO-standardized death rate from this disease in Taiwan was 14.9% (5,687 deaths)^3^. These statistics reflect its great impacts on human health and the global mortality rate. Thus, a careful examination of the specific growth factors involved and their associations with metastasis of CRC and patient survival might help in the diagnosis, determining the prognosis, and eventually in reducing the death rate from CRC.

The vascular endothelial growth factor (VEGF) family, including VEGF-A, -B, -C, -D, and placental growth factor (PlGF), has been associated with the risk of CRC^4–16^. In particular, high VEGF-A expression positively correlated with stages II-IV or with reduced survival in Finish CRC patients^5^; while, bevacizumab, a recombinant humanized monoclonal antibody against VEGF-A, improved patient survival^6^. In a New Zealand study, VEGF-A and -C levels correlated with the tumor grade and tumor size of CRC, VEGF-B expression was decreased in carcinomas compared with adenomas, and levels of VEGF-D were higher in normal tissues than CRC tissues^7^. In addition, VEGF-C was higher in the CRC tissues of UK patients relative to normal colon epithelium and correlated with metastasis in Portugal CRC patients^8,9^; while, VEGF-D was an independent prognostic marker for survival in the UK CRC patients^10^. Although high levels of VEGF-C and VEGF-D proteins correlated with lymph node metastasis and the long-term prognosis in Chinese CRC patients^11^, there were no associations between other VEGF family members and the CRC stages. The fifth VEGF member PlGF positively correlated with CRC stage and patient survival in studies from Turkey and Korea^12,13^. In Taiwan, VEGF-A overexpression predicted early postoperative relapse and survival of stage I-III CRC patients^14^; while PlGF overexpression was higher in the advanced CRC group than the localized group and its serum levels were higher in preoperative CRC patients than healthy subjects^15,16^. The associations between other VEGF members and metastasis and poor survival are unknown in Taiwanese CRC patients. Together, clinical studies suggest that the association with CRC depends on the types and levels of the VEGFs, the patient’s clinicopathological variables, and the patient’s ethnicity. Little is known about VEGF protein profiles in tumor tissue vs. blood and their associations with metastasis and survival in CRC patients.

There are three main subtypes of VEGF receptors (VEGFRs), VEGFR-1, VEGFR-2, and VEGFR-3. Like their ligands, the receptors are associated with the clinicopathological variables of the CRC subjects and these associations vary with subtype and ethnicity^7–10,17–20^. For example, in a US study, VEGFR-1 was detected in primary CRC and hepatic metastases, but not in nonmalignant mucosa^18^; while, VEGFR-2 and VEGFR-3 correlated with invasive adenocarcinoma and hepatic metastasis, respectively, in Portugal CRC patients^9^. In a New Zealand study^7^, VEGFR-1 and VEGFR-2, but not VEGFR-3, correlated with lymph node involvement; while, in a UK study^10^, VEGFR-3 did not act as an independent prognostic marker for survival in CRC patients. It is worth exploring whether expression levels of the VEGFR members in tumor tissue were consistent with their soluble secreted isoforms in serum in distinguishing metastatic CRC (mCRC) from non-mCRC subjects.

The present study was designed to investigate both tumor tissue and circulating protein levels of the VEGF and VEGFR families in Taiwan CRC patients, and their associations with clinicopathological variables and survival. We also determined if the circulating VEGF and soluble VEGFR (sVEGFR) levels differed between healthy and CRC subjects.

## Results

### Clinicopathological characteristics

Among the 114 CRC patients screened at our clinic, 109 fulfilled the inclusion criteria and their clinicopathological characteristics are summarized in Table 1. The mean age of the subjects was 69.35 years and 77 men and 32 women were included. There were 60 (55%) and 49 patients (45%) with colon and rectal cancers, respectively. Thirty nine patients (36%) had a tumor that was larger than 5 cm, and 70 patients (64%) had a tumor with less than 5 cm. One hundred patients (92%) had well or moderately differentiated cancers, and 9 patients (8%) had poor or mucinous cancers. There were 50 cases of early-stage (I/II) and 59 cases of late-stage (III/IV) CRC. There were 55 patients (51%) with lymph node metastasis (N1/N2) and 46 patients (42%) with elevated serum carcinoembryonic antigen (CEA) levels >5 ng/ml. According to the BMI criteria for overweight and obese subjects in Taiwan (cut-off values of 24 and 27, respectively), there were five patients (4.6%) who were underweight, 60 patients (55%) of normal weight, 39 patients (35.8%) who were overweight, and five obese patients (4.6%).

**Table 1:** The percentage of CRC patients (n = 109) reporting a positive VEGF or VEGFR in tumor tissues varied with clinicopathological characteristics after analysis of immunohistochemistry.

Characteristics	n (%)	VEGF-A n (%)	VEGF-B n (%)	VEGF-C n (%)	VEGF-D n (%)	PlGF n (%)	VEGFR-1 n (%)	VEGFR-2 n (%)	VEGFR-3 n (%)
Gender									
male	77 (71)	65 (84)	61 (79)	60 (78)	62 (81)	62 (81)	55 (71)	66 (86)	60 (78)
female	32 (29)	31 (97)^†^	25 (78)	23 (72)	24 (75)	29 (91)	23 (72)	21 (66)*	23 (72)
Age, Mean years 69.35 ± 1.26 (34–93)									
<65	39 (36)	30 (77)	21 (54)	19 (49)	24 (62)	27 (69)	20 (51)	26 (67)	23 (59)
≧65	70 (64)	66 (94)*	65 (93)*	64(91)*	57 (81)*	64 (91)*	58 (83)*	61 (87)*	54 (77)*
BMI (Kg/m^2^)									
<24	65 (60)	57 (88)	49 (75)	48 (74)	38 (65)	49 (75)	37 (57)	48 (74)	37 (57)
≧24	44 (40)	39 (89)	37 (84)	35 (80)	33 (77)^†^	42(96)*	41 (93)*	39 (89)^†^	31 (71)^†^
Location									
colon	60 (55)	50 (83)	43 (72)	38 (63)	43 (72)	48 (80)	38 (63)	51 (85)	51 (85)
rectum	49 (45)	46 (94)^†^	43 (88)*	45 (92)*	42 (86)^†^	43 (88)	40 (82)*	36 (74)^†^	32 (65)*
Size (cm)									
<5	70 (64)	65 (93)	61 (87)	52 (74)	48 (69)	59 (84)	45 (64)	51 (73)	49 (70)
≧5	39 (36)	31 (80)*	25 (64)*	31 (80)	30 (77)	32 (82)	33 (85)*	36 (92)*	34 (87)*
Histological differentiation									
well/moderate	100 (92)	88 (88)	78 (78)	75 (75)	73 (73)	83 (83)	70 (70)	79 (79)	70 (70)
poor/mucinous	9 (8)	8 (89)	8 (89)	8 (89)	8 (89)	8 (89)	8 (89)	8 (89)	8 (89)
UICC TNM stage									
I/II (non-metastasis)	50 (46)	39 (78)	35 (70)	30 (60)	31 (78)	37 (74)	31 (62)	34 (68)	32 (64)
III/IV (metastasis)	59 (54)	54 (92)*	51 (86)*	53 (90)*	50 (85)*	54 (92)*	47 (80)*	53 (90)*	49 (83)*
Lymph node metastasis									
N0	54 (49)	51 (94)	47 (87)	36 (67)	37 (69)	42 (78)	32 (59)	43 (80)	35 (65)
N1/N2	55 (51)	45 (82)*	39 (71)*	47 (86)*	45 (82)^†^	49 (89)^†^	46 (84)*	44 (80)	47 (86)*
CEA (ng/ml)									
<5	63 (58)	57 (91)	51 (81)	45 (71)	42 (67)	51 (81)	40 (64)	48 (76)	45 (71)
≧5	46 (42)	39 (85)	35 (76)	38 (83)	33 (72)	40 (87)	38 (83)*	39 (85)	37 (80)

Notes: The percentage of cytoplasmic staining of tumor cells for VEGF or VEGFR more than 25% was considered as “positive”. The percentage of patients reported with a positive VEGF or VEGFR in tumor tissues per number (n) of the variable patients. The mean age with a range from 34 to 93 years old was estimated from 109 CRC patients. The means ± SD values for CEA less than 5 ng/ml and more than 5 ng/ml were 2.87 ± 0.16 ng/ml and 56.82 ± 22.08 ng/ml, respectively. VEGF, vascular endothelial growth factor; VEGFR, VEGF receptor; CRC, colorectal cancer; BMI, body mass index; CEA, carcinoembryonic antigen. ^†^*p* < 0.1; *p < 0.05.

### Tissue level of VEGF proteins

**Figure 1:**
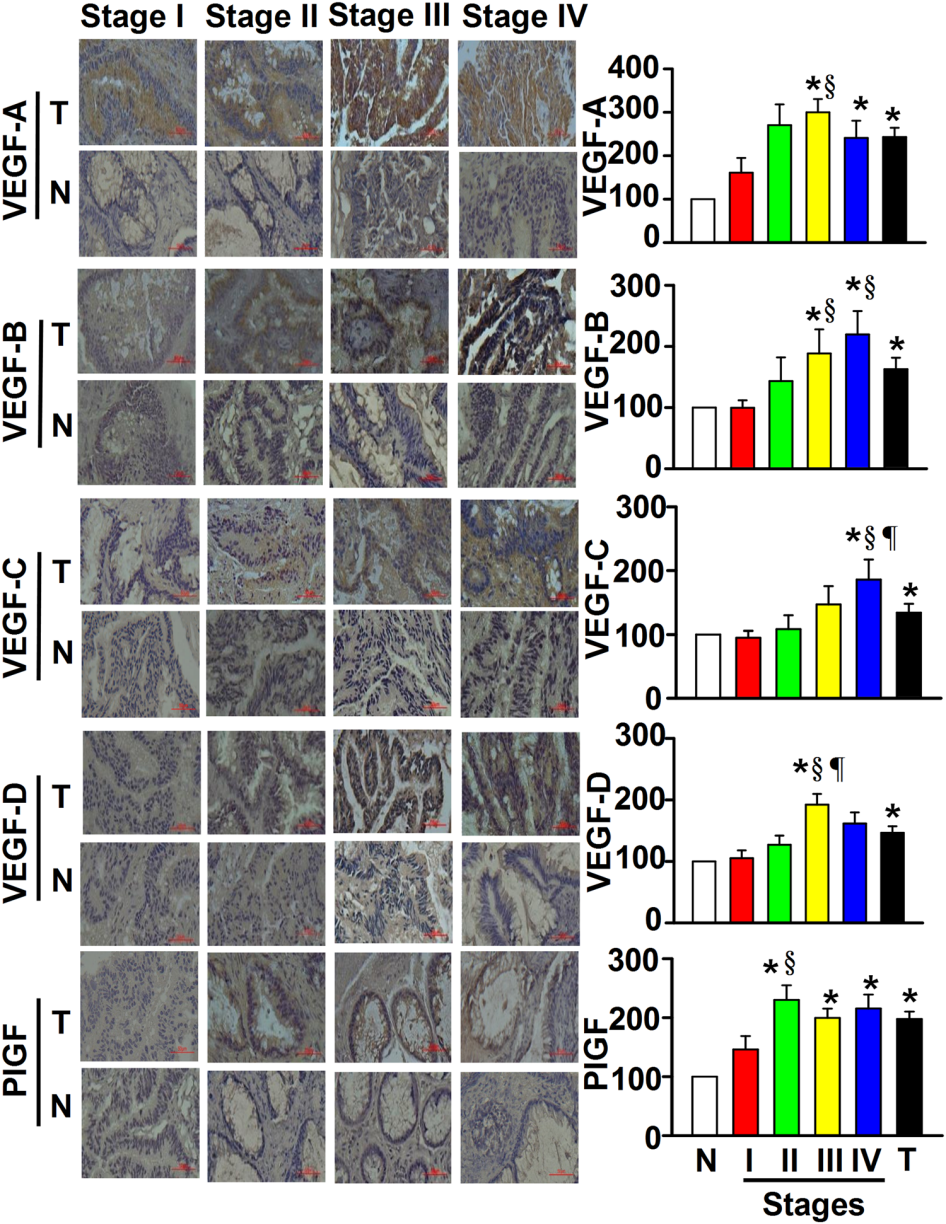
Differential protein levels of the vascular endothelial growth factor (VEGF) family were found between tumor tissues (T) and normal adjacent epithelium (N) when colorectal cancer (CRC) was examined by the immunohistochemical staining method. With an Image-J system, each scanned T or N section with 3,3′-diaminobenzidine (DAB)-staining intensity was quantified within 5 regions of interest, the average integrated optical density (IOD) was calculated, and data were normalized to N according to the IOD values. After normalization to N of each given stage of the CRC, the levels of VEGF and placental growth factor (PlGF) proteins were expressed as a percentage of normal adjacent epithelium, and data were presented as means ± SD. Data of VEGF and PlGF shown here for tumor (T) patients (X-axis) of the bar graph were expressed as means ± SD from all 109 CRC patients with stages I, II, III and IV. *P < 0.05, tumor tissues *vs* normal adjacent epithelium; ^§^*P* < 0.05, tumor patients *vs* Stage I; ^¶^*P* < 0.05, tumor patients *vs* Stage II. Magnification, 40*X*.

Levels of VEGF proteins differed between tumor tissues and normal adjacent epithelium (NA^E^) (Fig. 1). When tumor tissues were stage-specifically compared with NA^E^, the following were significantly higher: VEGF-A in all stages, VEGF-B in stages III-IV, VEGF-C in stage IV, VEGF-D in stage III, and PlGF in stages II-IV (Fig. 1). Stage III tumors had significantly higher VEGF-A, -B, and -D levels than stage I, while stage IV had higher VEGF-B and -C proteins than stage I. Additionally, VEGF protein levels varied according to other clinicopathological features of patients, independent of gender and CEA values (Table 1). For example, all the VEGFs in tumor tissue were higher in CRC patients aged ≥65 than in those aged <65. There were higher percentages of overweight and obese CRC patients positive for PlGF protein than normal weight and underweight CRC patients. A greater percentage of rectum tumor patients were positive for VEGF-B or VEGF-C protein in tumor tissues than colon tumor patients. About 80% and 64% of patients with a tumor size ≥5 cm had VEGF-A-positive and VEGF-B-positive tumor tissues, respectively, compared with 93% and 87% of patients with a tumor size <5 cm. All VEGFs were significantly higher when comparing mCRC with non-mCRC (Table 1). There were also significantly increased VEGF-A, VEGF-B, and VEGF-C levels, as well as a trend towards increased VEGF-D and PlGF proteins (Table 1), in patients with lymph node metastasis vs. those without lymph node metastasis.

### Tissue levels of VEGFR proteins

**Figure 2:**
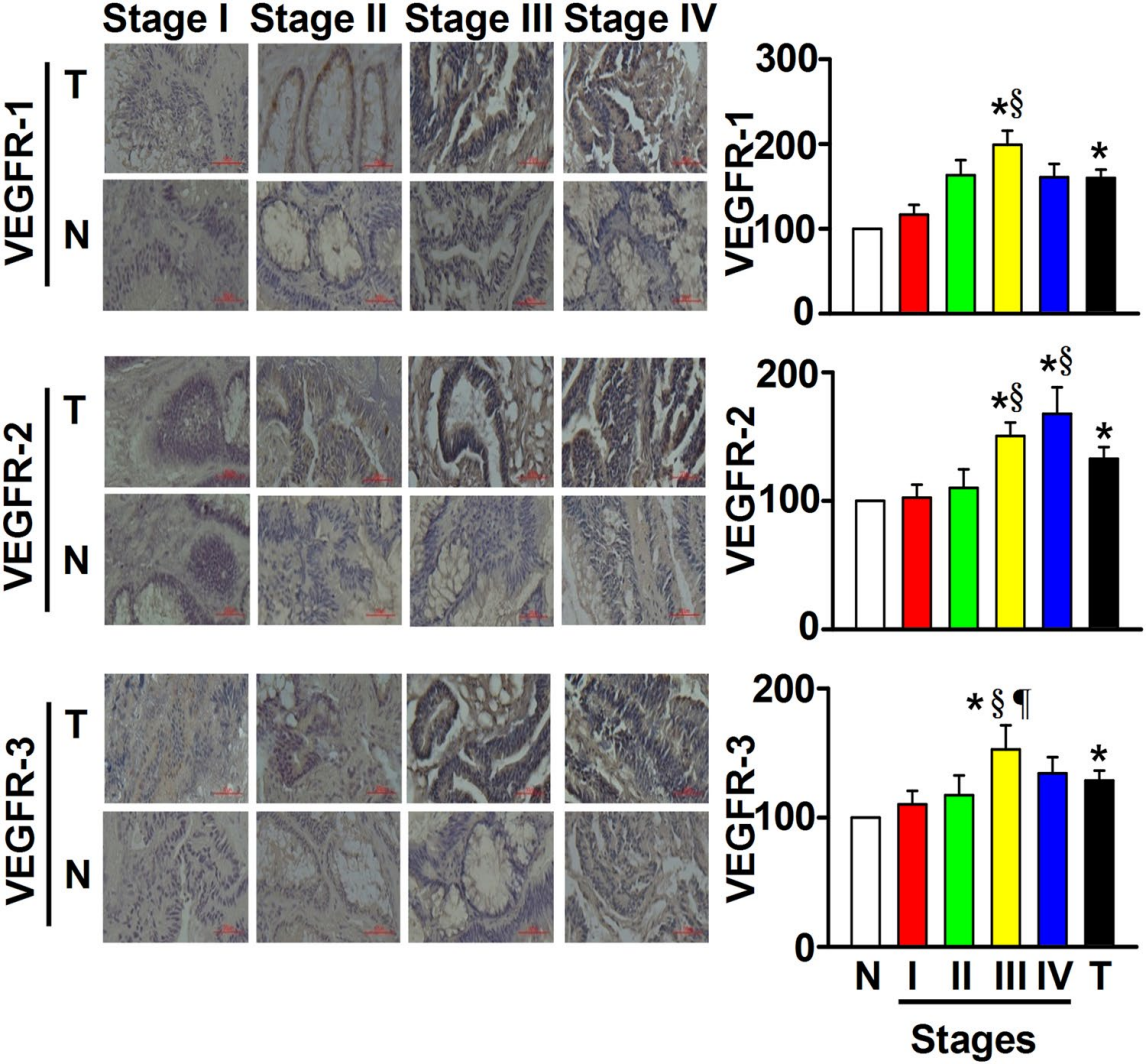
Differential protein levels of the vascular endothelial growth factor receptor (VEGFR) family were found between tumor tissues (T) and normal adjacent epithelium (N) when colorectal cancer (CRC) was examined by the immunohistochemical staining method. With an Image-J system, each scanned T or N section with 3,3′-diaminobenzidine (DAB)-staining intensity was quantified within 5 regions of interest, the average integrated optical density (IOD) was calculated, and data were normalized to N according to the IOD values. After normalization to N of each given stage of the CRC, the levels of VEGFR proteins were expressed as a percentage of normal adjacent epithelium, and data were presented with as means ± SD. Data of VEGFR shown here for tumor (T) patients (X-axis) of the bar graph were expressed as means ± SD from all 109 CRC patients with stages I, II, III and IV. *P < 0.05, tumor tissues *vs* normal adjacent epithelium; ^§^*P* < 0.05, tumor patients *vs* Stage I; ^¶^*P* < 0.05, tumor patients *vs* Stage II. Magnification, 40*X*.

Like their ligands, all three VEGFRs had different protein levels when comparing tumor tissues with NA^E^ and mCRC with non-mCRC (Fig. 2). Tumor tissues had higher VEGFR levels than NA^E^; particularly, VEGFR-1 and -3 proteins were significantly higher in stage III and VEGFR-2 was significantly higher in stages III-IV. Additionally, stage III patients had higher levels of all VEGFRs than stage I or stage II patients. There were also significant differences in the VEGFRs according to gender, age, body weight, tumor site, tumor size, lymph node metastasis, or CEA values (Table 1). About 86% of male CRC patients had VEGFR-2-positive tumor tissues compared with 66% of female CRC patients, and this difference was significant. About 83%, 87%, and 77% of CRC patients aged ≥65 had VEGFR-1-positive, VEGFR-2-positive, and VEGFR-3-positive tumor tissues and this was significantly more than the 51%, 67%, and 59% of CRC patients aged <65. Among overweight and obese CRC patients, 93% had VEGFR-1-positive tumor tissues compared with 57% of normal and underweight CRC patients. There were 82% and 65% of rectum tumor patients and 63% and 85% of colon tumor patients positive for VEGFR-1 and VEGFR-3 protein in tumor tissues, respectively. About 85%, 92%, and 87% of patients with a tumor size ≥5 cm had VEGFR-1-positive, VEGFR-2-positive, and VEGFR-3-positive tumor tissues compared with 64%, 73%, and 70% of patients with a tumor size <5 cm. The VEGFR-1, VEGFR-2, and VEGFR-3 proteins were all significantly higher when there was metastasis compared with non-metastasis, according to the TNM system (Table 1). However, about 84% and 86% of patients with lymph node metastasis were positive for VEGFR-1 and VEGFR-3 proteins in tumor tissues, respectively, compared with 59% and 65% of patients without lymph node metastasis. About 83% of patients with serum CEA levels ≥5 ng/ml had VEGFR-1-positive tumor tissues compared with 64% of patients with serum CEA levels <5 ng/ml.

### Serum VEGF levels in healthy and CRC subjects

**Figure 3:**
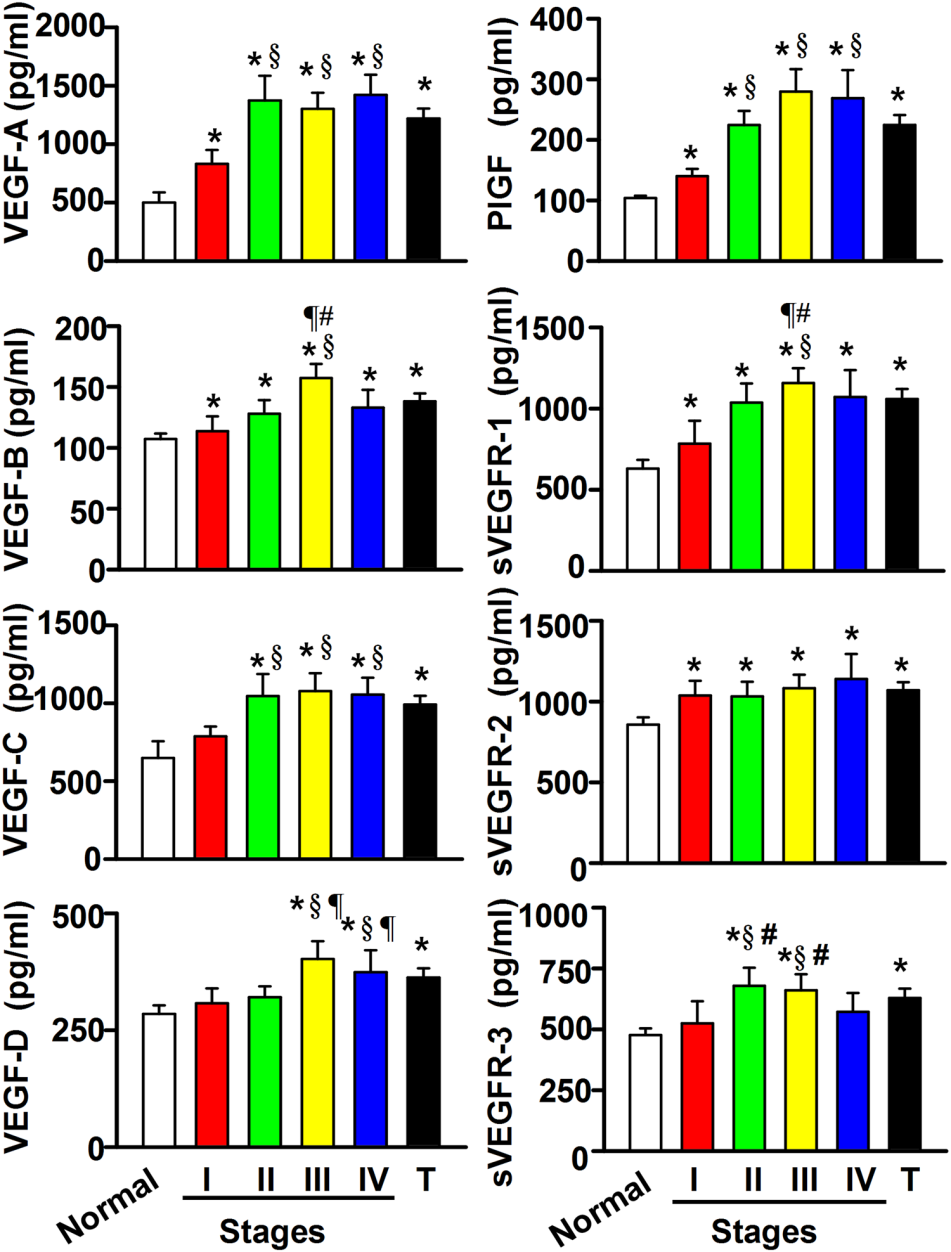
Differences in the circulating level of the vascular endothelial growth factor (VEGF) family and the soluble secreted VEGF receptor (sVEGFR) family were found among healthy subjects and different stages of the colorectal cancer patients after the analysis of ELISA. Data were expressed as means ± SD. Data of tumor (T) patients (X-axis) were expressed as means ± SD (n = 80) from all CRC patients with stages I, II, III and IV. ^*^P < 0.05, tumor patients *vs* normal subjects; ^§^*P* < 0.05, tumor patients *vs* Stage I; ^¶^*P* < 0.05, tumor patients *vs* Stage II; ^#^*P* < 0.05, tumor patients *vs* Stage IV.

Before we examined if the levels of circulating VEGF proteins were similar to their tumor tissue levels in their associations with different stages of CRC, we compared CRC patients with healthy subjects (Fig. 3). The CRC patients had higher circulating VEGF levels than healthy subjects. We found VEGF-A, -B, and PlGF proteins were significantly higher at all stages; while, serum VEGF-C and -D proteins were significantly higher in stages II-IV and III-IV, respectively (Fig. 3). The following serum levels were generally higher compared with stage I: VEGF-A in stages II-IV, VEGF-B in stage III, VEGF-C in stages II-IV, VEGF-D in stages III-IV, and PlGF in stages II-IV. Patients with mCRC had higher circulating levels of VEGF-A, -B, -C, -D, and PlGF proteins than those without metastasis (Table 2). Also, the serum VEGF proteins varied with gender, age, body weight, tumor site, tumor size, tumor differentiation, TNM stage, or lymph node metastasis.

**Table 2:** Serum levels of VEGF and sVEGFR proteins varied with clinicopathological characteristics of CRC patients (n = 80) after ELISA analysis

Characteristics	Serum (pg/ml)								
	n (%)	VEGF-A	VEGF-B	VEGF-C	VEGF-D	PlGF	sVEGFR-1	sVEGFR-2	sVEGFR-3
Gender									
male	53 (66)	1248 ± 28	142 ± 24	998 ± 57	383 ± 25	281 ± 38	1080 ± 42	1060 ± 38	621 ± 31
female	27 (34)	931 ± 88*	138 ± 17	986 ± 32	375 ± 16	209 ± 14*	1028 ± 52	1099 ± 43	595 ± 51
Age, Mean years 67.63 ± 1.56 (34–93)									
<65	27 (34)	912 ± 28	142 ± 13	782 ± 57	372 ± 26	207 ± 17	1071 ± 59	1025 ± 60	643 ± 33
≧65	53 (66)	1354 ± 33*	153 ± 20	1322 ± 80*	359 ± 26	248 ± 33*	1099 ± 68	1033 ± 71	632 ± 50
BMI (Kg/m^2^)									
<24	50 (63)	1235 ± 82	142 ± 16	937 ± 62	387 ± 32	267 ± 21	1070 ± 43	996 ± 58	613 ± 40
≧24	30 (37)	1286 ± 44	145 ± 21	1315 ± 106*	370 ± 28	201 ± 16*	1051 ± 53	1090 ± 48	630 ± 48
Location									
colon	49 (61)	923 ± 32	112 ± 14	925 ± 59	395 ± 44	246 ± 18	1179 ± 43	1068 ± 33	712 ± 62
rectum	31 (39)	1318 ± 87*	150 ± 23*	1530 ± 54*	389 ± 33	232 ± 23	695 ± 64*	687 ± 43*	585 ± 48*
Size (cm)									
<5	56 (70)	933 ± 42	140 ± 23	916 ± 53	348 ± 38	300 ± 21	988 ± 52	1021 ± 42	625 ± 35
≧5	24 (30)	1302 ± 35	144 ± 19	1215 ± 41*	432 ± 52*	216 ± 15*	1066 ± 48	1009 ± 53	638 ± 53
Histological differentiation									
well/moderate	73 (91)	1215 ± 86	105 ± 27	979 ± 56	383 ± 31	235 ± 14	842 ± 51	733 ± 46	593 ± 43
poor/mucinous	7 (9)	1664 ± 104*	148 ± 25*	1256 ± 62*	476 ± 44*	305 ± 26*	1136 ± 68*	1098 ± 61*	723 ± 52*
TNM stage									
I/II (non-metastasis)	35 (44)	1048 ± 64	124 ± 19	901 ± 79	329 ± 38	182 ± 15	952 ± 70	1034 ± 66	627 ± 65
III/IV (metastasis)	45 (56)	1385 ± 77*	151 ± 28*	1131 ± 80*	405 ± 30*	275 ± 21*	1131 ± 81	1102 ± 75	629 ± 56
Lymph node metastasis									
N0	39 (49)	1201 ± 32	119 ± 21	934 ± 48	341 ± 46	209 ± 14	1057 ± 49	1056 ± 47	625 ± 55
N1/N2	41 (51)	1305 ± 58^†^	152 ± 28*	1570 ± 81*	439 ± 60*	279 ± 20*	1069 ± 62	1089 ± 53	634 ± 63
CEA (ng/ml)									
<5	43 (54)	1319 ± 129	142 ± 18	1025 ± 83	386 ± 31	238 ± 18	1074 ± 52	1099 ± 77	618 ± 74
≧5	37 (46)	1080 ± 101^†^	148 ± 24	978 ± 58	372 ± 41	245 ± 22	1108 ± 62	993 ± 62	599 ± 87

Notes: VEGF, vascular endothelial growth factor; sVEGFR, soluble VEGF receptor; CRC, colorectal cancer; ELISA, enzyme-linked immunosorbent assay; BMI, body mass index; CEA, carcinoembryonic antigen. The mean age with a range from 34 to 93 years old was estimated from 80 CRC patients. The means ± SD values for CEA less than 5 ng/ml and more than 5 ng/ml were 2.95 ± 1.29 ng/ml and 69.19 ± 27.94 ng/ml, respectively. ^†^*p* < 0.1; *p < 0.05.

### Serum sVEGFR levels in healthy and CRC subjects

All three VEGFRs may be membrane-bound or alternatively spliced into sVEGFRs that are able to compete for VEGF binding with either membrane-bound receptors or monoclonal antibody-based drugs^6,17^. Here, we found that the levels of circulating sVEGFR proteins differed between CRC and healthy subjects and with CRC stage (Fig. 3). When CRC patients were compared with healthy subjects, serum levels of sVEGFR-1 and -2 proteins were significantly higher in all stages and sVEGFR-3 was significantly higher in stages II-III. Stage III patients also had higher sVEGFR-1 and -3 than stage I patients. Although there were no significant differences in the levels of all sVEGFR proteins in mCRC vs. non-mCRC patients (Table 2), all sVEGFR proteins varied with tumor sites and differentiation.

### Correlation of VEGF with VEGFR in CRC

The correlations between VEGFs and VEGFRs in the CRC subjects are summarized in Table 3. We observed a positive correlation between tumor tissue and serum levels of the individual VEGF proteins. Also VEGFR-1 positively correlated with sVEGFR-1, VEGFR-2 with sVEGFR-2, and VEGFR-3 with sVEGFR-3. In addition, there were positive correlations between VEGFR-1 and serum VEGF-A, -B, and PlGF; between VEGFR-2 and serum VEGF-A, -C, and –D; between VEGFR-3 and VEGF-C and -D; between VEGF-A and VEGF-B; and between VEGF-C and VEGF-D proteins.

**Table 3:** Pearson correlation coefficients between tumor tissue and serum levels of VEGF and VEGFR proteins in the CRC patients (n = 80) after analyses of IHC and ELISA.

Tumor tissues	Serum							
	VEGF-A	VEGF-B	VEGF-C	VEGF-D	PlGF	sVEGFR-1	sVEGFR-2	sVEGFR-3
VEGF-A	0.462**	0.297**	0.215	0.107	0.248	0.384*	0.547***	0.161
VEGF-B	0.314**	0.318**	0.224	0.157	0.251	0.414***	0.231	0.175
VEGF-C	0.187	0.157	0.522**	0.360**	0.181	−0.112	0.376**	0.412***
VEGF-D	0.156	0.188	0.441**	0.417**	0.181	0.137	0.295*	0.410***
PlGF	0.231	0.205	0.230	−0.087	0.311*	0.384**	−0.229*	0.117
VEGFR-1	0.348*	0.585**	0.127	0.144	0.432*	0.390*	0.176	0.187
VEGFR-2	0.874***	0.241	0.612**	0.375*	−0.047	−0.018	0.299*	0.194
VEGFR-3	0.201	0.096	0.579**	0.714***	0.194	0.211	0.231	0.345*

Notes: CRC, colorectal cancer; VEGF, vascular endothelial growth factor; VEGFR, VEGF receptor; sVEGFR, soluble VEGFR; IHC, immunohistochemistry; ELISA, enzyme-linked immunosorbent assay. *p < 0.05; **p < 0.01; ***p < 0.001; −, negative correlation.

### Association of VEGF and its receptors with survival and prognosis in CRC

**Figure 4:**
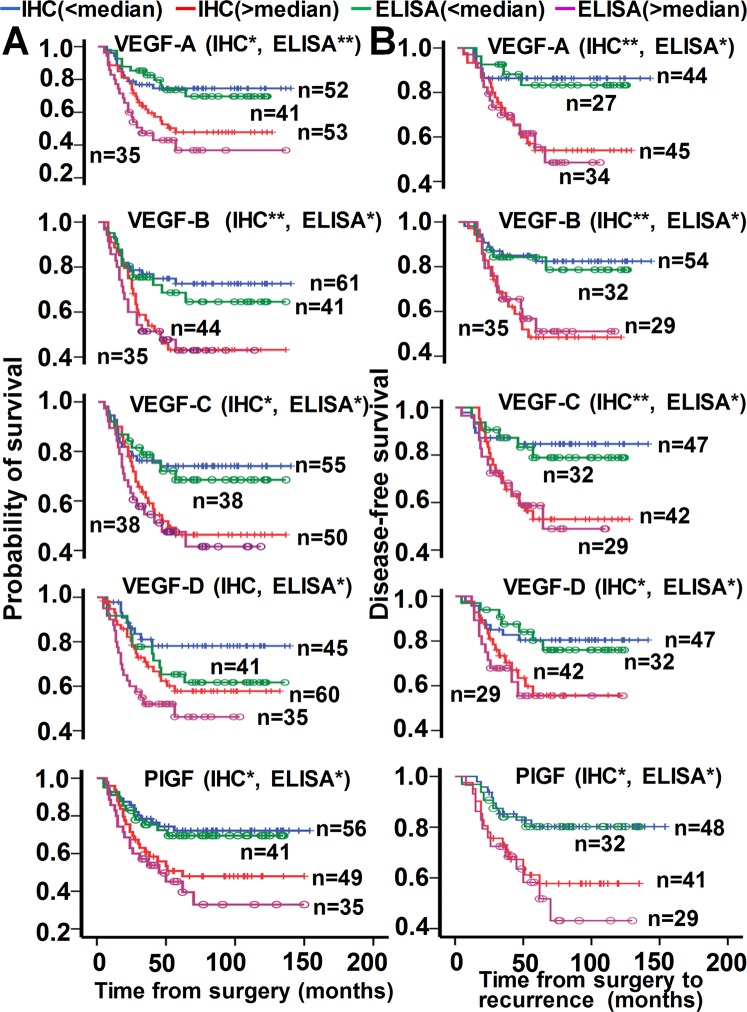
Impact of VEGF protein expression on patient survival. Kaplan-Meier survival analysis indicating cause-specific overall survival (**A**) and disease-free survival (**B**) of patients after surgery for CRC, according to grade of VEGF expression as described in the section “Results”: blue and green lines, low grade (<median); red and pink lines, high grade (>median); +, censored observations; blue and red lines, IHC data; green and pink lines, ELISA data; *p < 0.05; **p < 0.01; N, sample number. The IHC presented here indicated VEGF or PlGF detected in tumor tissues, while the ELISA indicated VEGF or PlGF detected in serum.

**Figure 5:**
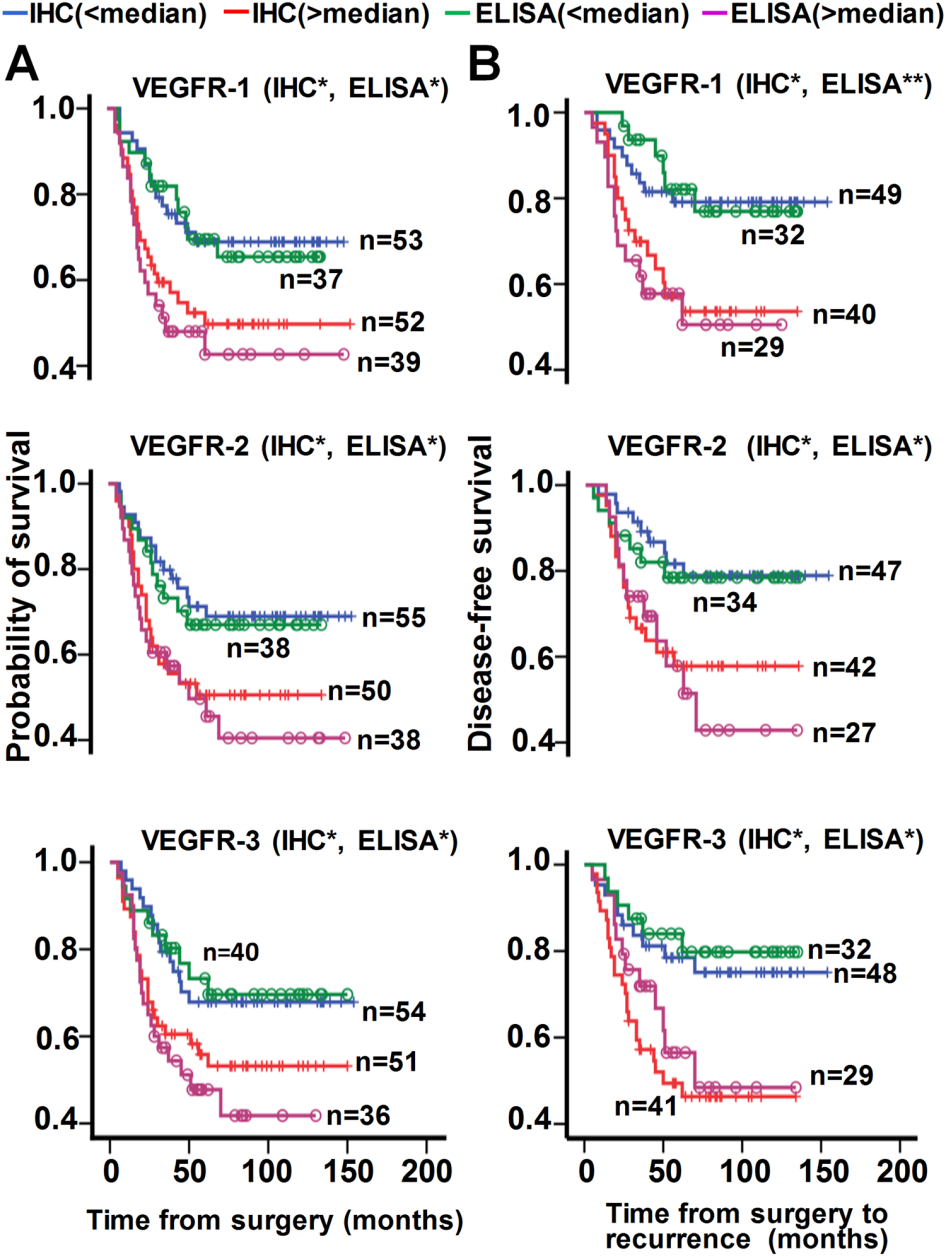
Impact of VEGFR protein expression on patient survival. Kaplan-Meier survival analysis indicating cause-specific overall survival (**A**) and disease-free survival (**B**) of patients after surgery for CRC, according to grade of VEGFR and sVEGFR expression as described in the section “Results”: blue and green lines, low grade (<median); red and pink lines, high grade (>median); +, censored observations; blue and red lines, IHC data; green and pink lines, ELISA data; *p < 0.05; **p < 0.01; N, sample number. The IHC presented here indicated VEGFR detected in tumor tissues, while the ELISA indicated sVEGFR detected in serum.

Patients were separated into two groups according to high-grade (>median) or low-grade (<median) VEGF, VEGFR, and sVEGFR expression. Patients with high-grade or low-grade levels of VEGF, PlGF, VEGFR, and sVEGFR were defined as those observed with above or below of their respective median values (Supplementary Table S1). Patients with high-grade levels of VEGF-A, VEGF-B, VEGF-C, VEGF-D, PlGF, VEGFR-1, VEGFR-2, VEGFR-3, sVEGFR-1, sVEGFR-2, and sVEGFR3 generally had a shorter overall survival (Figs 4A, 5A) and a shorter disease-free survival (Figs 4B, 5B) compared with those with low-grade expression. The median overall survival and disease-free results are shown in Supplementary Tables S2, S3.

## Discussion

The VEGF family and its receptors act as modulators and biomarkers in CRC, based on previous studies^5–20^. The present study described the synchronous protein expression of all members of the VEGF and VEGFR families in CRC, and the results provide a better understanding of the correlation of each family member with the clinicopathological variables of Taiwanese CRC patients. The changes in VEGF levels in CRC patients were synchronous in tumor tissue and serum. The findings also showed that patients with stages III and IV mCRC had higher VEGF-A, -B, -C, -D, and PlGF levels in tumor tissues and serum than those with stages I and II non-mCRC. Although the mCRC patients had higher VEGFR proteins in tumor tissue and similar circulating sVEGFR levels than non-mCRC patients, the tumor tissue VEGFR positively correlated with the serum sVEGFR, which may indicate that changes in the circulating sVEGFR levels resulted from the secretion of tumor tissue. The assumption is also indirectly supported by the previous finding that all three VEGFRs can be spliced into soluble secreted VEGFRs in blood^17^ and that mCRC patients exhibited higher serum sVEGFR levels than healthy subjects.

Different subtypes of VEGFRs not only selectively mediate cellular responses to VEGF, but also have a clinical association with CRC that depends on the VEGFR isoforms and ethnic groups included in the study^17,21^. In particular, VEGFR-1 binds VEGF-A, -B, and PlGF to regulate the angiogenesis and epithelial-to-mesenchymal transition^21^, VEGFR-2 binds VEGF-A, -C, and -D to promote growth and migration of endothelial cells that are favorable for vascular permeability^22,23^, and VEGFR-3 binds VEGF-C and -D to regulate lymphangiogenesis^24,25^. The specific relationships between VEGF members and VEGFR isoforms in CRC were supported by our findings of positive correlations of VEGFR-1 with VEGF-A, -B, and PlGF; VEGFR-2 with VEGF-A, -C, and –D; and VEGFR-3 with VEGF-C and -D. The variety of interaction mechanisms may explain how advanced stage CRC cancer cells grow through all layers of the colon or rectum or spread to nearby tissues and distant organs with angiogenesis dependency. This notion is also supported by our findings that the CRC patients had higher protein levels of all VEGFs and VEGFRs in tumor tissues than non-tumor tissues and that patients in stages III/IV had higher VEGFs and VEGFRs levels in tumor tissues than stages I and II patients (Table 1). In addition, patients with mCRC and non-mCRC had higher circulating levels of VEGFs, PlGF, and sVEGFR than normal subjects.

Monoclonal antibody-based therapy against VEGF-A, PlGF, or VEGFR-2 has been effectively used in combination with chemotherapy for the treatment of mCRC to improve survival rates in patients^14,26–32^. For example, antiangiogenic therapy with bevacizumab plus FOLFIRI (Folinic acid/5-Fluorouracil/Irinotecan) to target VEGF-A is an effective and well-tolerated first-line treatment for mCRC^30,31^. Nonetheless, the overall impact of bevacizumab in prolonging survival has been limited^14^. While the survival has improved to 24- to 28-months, the overall prognosis of mCRC remains poor, with the 5-year survival generally between 5% and 8%, despite the availability of such therapies^14^. Since bevacizumab only targets VEGF-A, the presence of high levels of VEGFR-1 and -2 proteins in tumor tissue and their circulating sVEGFR proteins may compete with bevacizumab for VEGF-A binding, resulting in drug resistance. As the VEGF-B, VEGF-C, VEGF-D, and PlGF proteins can compete with VEGF-A for the VEGFR-1 or VEGFR-2 binding, the rising or retention levels of the four VEGF members during combinatorial therapy with bevacizumab and FOLFIRI might also compensate for the loss of VEGF-A. This may affect the effectiveness of drug treatment and tumor recurrence after surgery. In this study, we found that preoperative mCRC patients exhibited higher levels of all VEGF family members in blood and tumor tissue, had higher VEGFR proteins in tumor tissues, and retained the same level of sVEGFR in serum compared with non-mCRC patients. In parallel, higher circulating levels of sVEGFR-1 were present in stage III CRC patients than stage I; while, the sVEGFR-2 levels were the same in advanced stages. Moreover, we observed the following positive correlations: VEGFR-1 with VEGF-A, -B and PlGF; VEGFR-2 with VEGF-A, -C, and –D; and VEGF-A with VEGF-B, sVEGFR-1, and sVEGFR-2. Notably, when the five post-operative CRC patients treated with a combination of bevacizumab with FOLFIRI were examined (Supplementary Fig. S1), they exhibited lower circulating VEGF-A levels during the 12 cycles of treatment. Interestingly, VEGF-B and PlGF proteins were increased, VEGF-C, VEGF-D, and sVEGFR-1 proteins were maintained, and sVEGFR-2 was significantly decreased. Further research is necessary to determine whether alterations in the patterns of serum VEGF and sVEGFR protein levels caused by combinatorial therapy with bevacizumab and FOLFIRI will affect the effectiveness of drug treatment and tumor recurrence after surgery.

Association of VEGF expression with CRC has been reported to vary with the types and levels of the VEGFs, the patient’s clinicopathological variables, and the patient’s ethnicity^4–16,33–38^. Our experiments showed that Taiwanese patients with stages III and IV mCRC had higher VEGF-B and -D levels in tumor tissues than those with stages I and II non-mCRC and that patients with high-grade levels of VEGF-B and -D had a shorter overall survival and a shorter disease-free survival compared with those with low-grade expression. Like VEGF-A, VEGF-C and PlGF, both VEGF-B and VEGF-D could be prognostic markers for survival in Taiwanese CRC. This conclusion is consistent with those reported for high VEGF-B expression in carcinoma tissues in Belgian, German, and Japanese mCRC patients^33–35^, as well as those observed for positive associations of high VEGF-D expression with poor survival, lymph node metastasis, and progression of CRC in Belgian, Chinese, Japanese, and/or UK patients^10,11,33,36^. However, a New Zealand study for CRC patients indicated higher VEGF-B mRNA expression in adenoma than normal tissues and carcinomas, higher VEGF-D mRNA expression in normal tissues than tumor tissues, and no correlations of both VEGF-B and VEGF-D mRNA levels with the patient’s clinicopathological variables^7^. Notably, the IHC data from the same study showed low basal levels of VEGF-B and -D in histologically normal tissues, VEGF-B immunopositivity in both adenoma and invasive CRC, and VEGF-D immunopositivity in an invasive CRC. The observed VEGF-B and VEGF-D immunopositivity in the invasive CRC appears consistent with those observed for high VEGF-B and -D protein levels in mCRC of our study and other reports^10,11,33–36^. Unfortunately, the researchers did not take further steps to quantify VEGF-B and VEGF-D protein levels from New Zealand CRC patients in association with tumor stages and patient survival. Possible explanations for the discrepancy between studies are that the distinct isoforms of VEGF-B and VEGF-D occur in human normal tissues and CRC at varying levels and that primers and antibodies used for respective detections of VEGF-B and VEGF-D mRNAs and proteins vary with their spliced variants, isoforms, primer sequences, and/or antibody affinity employed^10,11,33–38^.

There were several limitations in this study. First, the analyses of VEGF and VEGFR protein levels have only been carried out in a single cohort without an independent validation cohort. Because of the limits of grant budgets and sample sizes, we did not perform an independent validation cohort study, or begin with a group of non-exposed individuals that developed the CRC or not in our follow-up study. Second, the cohort approach is time consuming and is prone to bias due to loss to follow-up over a long period^39^. Indeed, we had 29 CRC patients unwilling to participate in the ELISA study over the period, 4 CRC patients loss to follow-up because of the deaths with heart disease, and more than 15 stage IV CRC patients out of the prognosis of disease-free survival. Third, although we found level differences of serum VEGF and sVEGFR proteins between non-CRC and CRC subjects, in which the sera of non-CRC subjects were collected from healthy subjects who had undergone a colonoscopy study, we were not allowed to get the colon or rectal tissues from healthy subjects for our cohort study. Because of these methodological limitations, the interpretations of our results from a single cohort study should be cautious, and firm conclusions as to whether a causal relationship between VEGF, VEGFR, and Taiwanese CRC patients is established will require more thorough independent validation cohort studies on a large population basis.

We conclude that patients with mCRC have higher VEGF-A, -B, -C, -D, PlGF, VEGFR-1, -2, and -3 proteins in tumor tissues than non-mCRC patients, and higher circulating levels of VEGF-A, -B, -C, -D, and PlGF proteins. The tumor tissue levels were synchronous with circulating levels of VEGFs, as were the correlations with VEGFRs and sVEGFRs and relationships with clinicopathological variables. Thus, particular VEGF, VEGFR, and/or sVEGFR proteins may be useful markers for gauging the clinical effect of various treatments on CRC patients as high protein levels of VEGFs, VEGFRs, and sVEGFRs are associated with poor survival in CRC patients living in Taiwan.

## Methods

### Patients and samples

Between 2005 and 2017, a total of 114 CRC patients were enrolled from the Colorectal Section of the Tri-Service General Hospital (Taipei, Taiwan) and Taoyuan Armed Forces General Hospital. Written informed consent to analyze tumors and sera was obtained from the patients before surgery and all experimental protocols (TSGHIRB No. 098-05-292, 2-105-05-129, TY101-14, and TY102-08) in this study were approved by the Institutional Research Board (Ethical Committee) at the National Defense Medical Center of the Tri-Service General Hosptial (Taiwan). The methods were carried out in accordance with the relevant guidelines and regulations. Exclusion criteria included patients who had preoperative chemotherapy or radiotherapy and a family history of CRC or polyposis. We examined the clinical parameters of the CRC patients, including personal profiles, colonoscopic biopsy results, clinical staging, pathologic diagnosis, and lymph node metastasis^40,41^. Colonic tissues were collected from freshly isolated surgical resections from CRC patients, normal colonic mucosa was obtained from the distal edge of the resection at least 10 cm from the tumor, and tumor tissues were taken from the edge of the tumor with exclusion of grossly necrotic tissues. All tissues were snap-frozen in liquid nitrogen and stored at −80 °C until later use. Sera were collected at the time of examination before surgery. Among the 109 CRC patients fulfilling the inclusion criteria, 80 were also enrolled in the serum study. The clinicopathological characteristics for the 109 and 80 CRC patients are summarized in Tables 1 and 2, respectively. For each clinicopathological variable, the ratio between the two groups was similar in tumor tissue and serum. Normal serum samples were also collected from 50 healthy subjects (mean age, 58.34 ± 1.53 years; and mean body mass index (BMI), 25.2 ± 2.4 kg/m^2^), with consent, who had undergone a colonoscopy study.

### Immunohistochemistry

We first used NOVA Histo (Cat#LB0200-0100, Bionovas biotechnology Co., Toronto, Ontario, CA) for the deparaffinization, rehydration, and antigen retrieval of formalin-fixed and paraffin-embedded sections (3 µm). Second, we followed the protocols for the EXPOSE Mouse and Rabbit Specific HRP/DAB detection IHC kit (Cat#ab80436, Abcam Plc. Cambridge, UK) to perform the immunohistochemical staining of VEGF and VEGFR proteins at room temperature. Sections were treated with 0.3% H_2_O_2_ for 10 min to neutralize endogenous peroxidase activity. They were washed twice with 1X PBST buffer and then blocked with Protein Block for 10 min. Primary VEGF and VEGFR antibodies were diluted as indicated in Supplementary Table S4 and incubated on the sections for 30 min. Sections were washed twice with buffer, incubated with Complement for 10 min, exposed to goat anti-HRP conjugate for 15 min, and visualized with the addition of Chromogen-DAB Substrate solution for 5 min. They were washed with buffer and then the nucleus was stained with hematoxylin for 3 min. The intensity of the immunostaining sections was graded as described by Vahedi *et al*.^42^ and quantitated using the image-J system^43^: 0, positive staining in less than <5% of cells; 1, 5–25% immunoreactive cells; 2, 26–50% immunoreactive cells; and 3, >50% immunoreactive cells. For statistical purposes, only immunoreaction final scores of 2 and 3 were considered positive. Native controls were performed in each stage of CRC patients omitting the primary antibody. Tissue sections and images of all of the CRC specimens were reassessed by a pathologist (Dr. J.-L. Chang) to confirm presence and grade of tumor.

### Enzyme-linked immunosorbent assay (ELISA)

Commercially available Quantikine ELISA kits for VEGF, sVEGFR, and carcinoembryonic antigen (CEA) were used according to the manufacturer’s instructions (Supplementary Table S5)^44^. The antibody specificity was shown in the instruction of the individual ELISA kit as described in detail previously by the manufacturers.

### Statistical analysis

Data were expressed as means ± SD, unless otherwise noted. We followed previous methods to analyze the results using SPSS statistical software (IBM SPSS Statistics 20.0.1)^45^. The unpaired Student’s t-test was used to examine differences between two groups. One-way ANOVA followed by the Student-Newman-Keuls multiple range test was used to examine differences among multiple groups. The Chi-square (χ^2^) test was used to analyze the relationship between the expression of VEGF in CRC with clinicopathological variables. The Pearson correlation analysis was used to examine correlations among VEGFs, VEGFRs, and sVEGFRs. Kaplan-Meier survival analysis was used to detect cause-specific overall survival and disease-free survival of patients after surgery for CRC. A *p*-value less than 0.05 was considered statistically significant.

## Supplementary information

Synchronous vascular endothelial growth factor protein profiles in both tissue and serum identify metastasis and poor survival in colorectal cancer

## Data Availability

All relevant data are within the paper and its Supplementary Information files.

## References

[1] Torre LA (2015). Global cancer statistics, 2012. CA Cancer J. Clin..

[2] World Health Organization Fact sheets: Cancer. http://www.who.int/mediacentre/factsheets/fs297/en/ (2017).

[3] Ministry of Health and Welfare, Taiwan. Taiwan Health and Welfare Report 2016. https://www.mohw.gov.tw/cp-137-521-2.html (2016).

[4] Korpanty G (2010). Molecular and clinical aspects of targeting the VEGF pathway in tumors. J. Oncol..

[5] Bendardaf R (2008). VEGF-1 expression in colorectal cancer is associated with disease localization, stage, and long-term disease-specific survival. Anticancer Res..

[6] Pavlidis ET, Pavlidis TE (2013). Role of bevacizumab in colorectal cancer growth and its adverse effects: a review. World J. Gastroenterol..

[7] Hanrahan V (2003). The angiogenic switch for vascular endothelial growth factor (VEGF)-A, VEGF-B, VEGF-C, and VEGF-D in the adenoma-carcinoma sequence during colorectal cancer progression. J. Pathol..

[8] Witte D (2002). Expression of the vascular endothelial growth factor receptor-3 (VEGFR-3) and its ligand VEGF-C in human colorectal adenocarcinoma. Anticancer Res..

[9] Martins SF (2013). Clinicopathological correlation and prognostic significance of VEGF-A, VEGF-C, VEGFR-2 and VEGFR-3 expression in colorectal cancer. Cancer Genomics Proteomics..

[10] White JD (2002). Vascular endothelial growth factor-D expression is an independent prognostic marker for survival in colorectal carcinoma. Cancer Res..

[11] Hu WG (2007). Vascular endothelial growth factors C and D represent novel prognostic markers in colorectal carcinoma using quantitative image analysis. Eur. Surg. Res..

[12] Kemık O (2012). Preoperative serum placenta growth factor level as a new marker for stage II or III colorectal cancer patients. Turk. J. Gastroenterol..

[13] Sung CY (2012). Expression of placenta growth factor in colorectal carcinomas. J. Korean Soc. Coloproctol..

[14] Tsai HL (2015). Decreased peritherapeutic VEGF expression could be a predictor of responsiveness to first-line FOLFIRI plus bevacizumab in mCRC patients. Int. J. Clin. Exp. Pathol..

[15] Wei SC (2005). Placenta growth factor expression is correlated with survival of patients with colorectal cancer. Gut..

[16] Wei SC (2009). Preoperative serum placenta growth factor level is a prognostic biomarker in colorectal cancer. Dis. Colon Rectum..

[17] Takahashi S (2011). Vascular endothelial growth factor (VEGF), VEGF receptors and their inhibitors for antiangiogenic tumor therapy. Biol. Pharm. Bull..

[18] Fan F (2005). Expression and function of vascular endothelial growth factor receptor-1 on human colorectal cancer cells. Oncogene..

[19] Yamaguchi T (2007). Overexpression of soluble vascular endothelial growth factor receptor 1 in colorectal cancer: association with progression and prognosis. Cancer Sci..

[20] Wei SC (2013). Flt-1 in colorectal cancer cells is required for the tumor invasive effect of placental growth factor through a p38-MMP9 pathway. J. Biomed. Sci..

[21] Fischer C, Mazzone M, Jonckx B, Carmeliet P (2008). FLT1 and its ligands VEGFB and PlGF: drug targets for anti-angiogenic therapy?. Nat. Rev. Cancer..

[22] Bates RC (2003). Flt-1-dependent survival characterizes the epithelialmesenchymal transition of colonic organoids. Curr. Biol..

[23] Holmqvist K (2004). The adaptor protein shb binds to tyrosine 1175 in vascular endothelial growth factor (VEGF) receptor-2 and regulates VEGF-dependent cellular migration. J. Biol. Chem..

[24] Dayanir V, Meyer RD, Lashkari K, Rahimi N (2001). Identification of tyrosine residues in vascular endothelial growth factor receptor-2/FLK-1 involved in activation of phosphatidylinositol 3-kinase and cell proliferation. J. Biol. Chem..

[25] Mäkinen T (2001). Isolated lymphatic endothelial cells transduce growth, survival and migratory signals via the VEGF-C/D receptor VEGFR-3. EMBO J..

[26] Wang JF, Zhang X, Groopman JE (2004). Activation of vascular endothelial growth factor receptor-3 and its downstream signaling promote cell survival under oxidative stress. J. Biol. Chem..

[27] Hurwitz H (2004). Bevacizumab plus irinotecan, fluorouracil, and leucovorin for metastatic colorectal cancer. N. Engl. J. Med..

[28] U. S. Food and Drug Administration. Center for Drug Evaluation and Research. Final Labeling Text, BL125085 Supplement, 2008.

[29] Wang K (2017). An apparent clinical pharmacokinetic drug-drug interaction between bevacizumab and the anti-placental growth factor monoclonal antibody RO5323441 via a target-trapping mechanism. Cancer Chemother. Pharmacol..

[30] Lee SJ (2017). Phase I trial and pharmacokinetic study of tanibirumab, a fully human monoclonal antibody to vascular endothelial growth factor receptor 2, in patients with refractory solid tumors. Invest. New Drugs..

[31] Kopetz S (2010). Phase II trial of infusional fluorouracil, irinotecan, and bevacizumab for metastatic colorectal cancer: efficacy and circulating angiogenic biomarkers associated with therapeutic resistance. J. Clin. Oncol..

[32] Chu E (2012). An update on the current and emerging targeted agents in meta-static colorectal cancer. Clin. Colorectal Cancer..

[33] Pringels S (2012). Clinical procedure for colon carcinoma tissue sampling directly affects the cancer marker-capacity of VEGF family members. BMC Cancer..

[34] Jayasinghe C, Simiantonaki N, Kirkpatrick CJ (2015). Cell type- and tumor zone-specific expression of pVEGFR-1 and its ligands influence colon cancer metastasis. BMC Cancer..

[35] Kawakami M (2003). Expression analysis of vascular endothelial growth factors and their relationsips to lymph node metastasis in human colorectal cancer. J. Exp. Clin. Cancer Res..

[36] Onogawa S (2004). Expression of VEGF-C and VEGF-D at the invasive edge correlates with lymph node metastasis and prognosis of patients with colorectal carcinoma. Cancer Sci..

[37] Bry M, Kivelä R, Leppänen V-M, Alitalo K (2014). Vascular endothelial growth factor-B in physiology and disease. Physiol. Rev..

[38] Stacker SA (1999). Biosynthesis of vascular endothelial growth factor-D involves proteolytic processing which generates non-covalent homodimers. J. Biol. Chem..

[39] Gordis, L. *Epidemiology*. (W.B. Saunders Company, London, 1996)

[40] Yeh CC (2016). Synchronous mucinous cystadenoma of the appendix in a patient with colon cancer. Transl. Cancer Res..

[41] Fleming M, Ravula S, Tatishchev SF, Wang HL (2012). Colorectal carcinoma: Pathologic aspects. J. Gastrointest. Oncol..

[42] Vahedi L (2015). Evaluation of VEGF immunohistochemical expression and correlation with clinicopathologic features in colorectal cancer. Govaresh..

[43] Schneider CA, Rasband WS, Eliceiri KW (2012). NIH Image to ImageJ: 25 years of image analysis”. Nat. Methods..

[44] Takahashi H (2016). Vascular endothelial growth factor (VEGF) concentration is underestimated by enzyme-linked immunosorbent assay in the presence of anti-VEGF drugs. Invest. Ophthalmol. Vis. Sci..

[45] Lee SW (2017). Suppressors of cytokine signaling in tuberculosis. PLoS One..

