# Supplementary material for: Synchronous vascular endothelial growth factor protein profiles in both tissue and serum identify metastasis and poor survival in colorectal cancer
Source: Sci Rep. 2019 Mar 12;9:4228. doi: 10.1038/s41598-019-40862-6 (PMC6414611; doi:10.1038/s41598-019-40862-6)
Supplement: Supplementary file 1 — Synchronous vascular endothelial growth factor protein profiles in both tissue and serum identify metastasis and poor survival in colorectal cancer [file 41598_2019_40862_MOESM1_ESM.pdf]

Supplementary Information

Synchronous vascular endothelial growth factor protein profiles in both tissue and serum identify metastasis and poor survival in colorectal cancer

Chien-Chih Yeh[1,2,3], Li-Jane Shih[3,4], Junn-Liang Chang[5], Yi-Wei Tsuei[6], Chang-Chieh Wu[3,7], Cheng-Wen Hsiao[3,7], Chih-Pin Chuu[8], and Yung-Hsi Kao[1,*]

[1]Department of Life Sciences, National Central University, Taoyuan 320, Taiwan

[2]Division of Colon and Rectal Surgery, Department of Surgery, Taoyuan Armed Forces General Hospital, Taoyuan 325, Taiwan

[3]National Defense Medical Center, Tri-Service General Hospital, Taipei 114, Taiwan

[4]Medical Laboratory, Taoyuan Armed Forces General Hospital, Taoyuan 325, Taiwan

[5]Department of Pathology and Laboratory Medicine, Taoyuan Armed Forces General Hospital, Taoyuan 325, Taiwan

[6]Department of Emergency, Taoyuan Armed Forces General Hospital, Taoyuan 325, Taiwan

[7]Division of Colon and Rectal Surgery, Department of Surgery, Tri-Service General Hospital, Taipei 114, Taiwan

[8]Institute of Cellular and System Medicine, National Health Research Institutes, Miaoli 350, Taiwan

[*] Address correspondence and send proofs to: Dr. Yung-Hsi Kao, Department of Life Sciences, National Central University, 300 Jhongda Road, Jhongli District, Taoyuan 320, Taiwan, Tel: (886)-3-4260839, FAX: (886)-3-4228482, E-mail: ykao@cc.ncu.edu.tw

Supplementary Table S1. Median values were used in survival statistics for definitions of CRC patients with low-grade or high-grade levels of VEGF, PlGF, and VEGFR proteins after analyses of IHC and ELISA

VEGF/VEGFR family members	Probability of survival		Disease-free survival	
	IHC(n=105)	ELISA(n=76)	IHC(n=89)	ELISA(n=61)
	median±SD	median±SD	median±SD	median±SD
VEGF-A	245± 45	894±115	269±41	873±128
VEGF-B	135± 34	121± 59	128±37	121± 57
VEGF-C	110± 52	866±135	104±50	858±117
VEGF-D	135± 47	338± 94	124±49	340± 74
PlGF	202± 56	222± 71	195±57	218± 85
VEGFR-1	153± 44	935±141	158±48	914±126
VEGFR-2	126± 41	946±109	122±33	943±124
VEGFR-3	127± 34	527± 89	127±37	536± 98

Notes: CRC, colorectal cancer; PlGF, placental growth factor; VEGF, vascular endothelial growth factor; VEGFR, VEGF receptor; IHC, immunohistochemistry; ELISA, enzyme-linked immunosorbent assay; low-grade, < median value of protein expression level in tumor tissue or serum; high-grade, > median value of protein expression level in tumor tissue or serum; SD, standard deviation. The VEGFR presented for ELISA represented the soluble VEGFR. The unit for the values of VEGF and VEGFR proteins analyzed by ELISA was the pg/ml. The values of VEGF and VEGFR proteins analyzed by IHC analysis represented a percentage of normal adjacent epithelium after the average integrated optical density in each sectioned tissue was scanned and calculated using the image-J system.

Supplementary Table S2. Median overall survival (OS) of months for CRC patients with low-grade or high-grade VEGF or VEGFR protein level in tumor tissues and serum after respective analyses of IHC and ELISA

Protein levels		IHC			ELISA		
		median *os* ±SE (months)	95% CI (months)	*p*-value	median *os* ±SE (months)	95% CI (months)	*p*-value
VEGF-A	low-grade	102±7	88-135	0.021	95±7	82-137	0.001
	high-grade	52±9	30-76		35±9	13-62	
VEGF-B	low-grade	98±9	81-129	0.008	96±6	66-125	0.046
	high-grade	48±11	21-98		50±11	12-87	
VEGF-C	low-grade	101±10	82-131	0.020	95±8	88-128	0.028
	high-grade	57±7	31-102		59±9	36-95	
VEGF-D	low-grade	85±9	78-127	0.066	84±5	54-103	0.040
	high-grade	67±12	41-119		68±12	36-97	
PlGF	low-grade	98±7	82-129	0.019	92±9	58-137	0.010
	high-grade	62±8	41-98		42±10	21-95	
VEGFR-1	low-grade	91±8	78-119	0.031	82±7	58-108	0.016
	high-grade	66±9	38-106		52±9	34-95	
VEGFR-2	low-grade	95±6	88-129	0.030	82±11	74-109	0.046
	high-grade	54±9	31-91		58±8	32-95	
VEGFR-3	low-grade	94±7	78-119	0.041	84±8	74-98	0.041
	high-grade	64±10	41-98		51±9	21-89	

Notes: CRC, colorectal cancer; VEGF, vascular endothelial growth factor; VEGFR, VEGF receptor; IHC, immunohistochemistry; ELISA, enzyme-linked immunosorbent assay; low-grade, < median value of protein expression level in tumor tissue or serum; high-grade, > median value of protein expression level in tumor tissue or serum; CI, confidence interval.

Supplementary Table S3. Median disease-free survival (DFS) of months for CRC patients with low-grade or high-grade VEGF or VEGFR protein level in tumor tissue and serum after respective analyses of IHC and ELISA

Protein levels		IHC			ELISA		
		Median *dfs* ±SE (months)	95% CI (months)	*p*-value	Median *dfs* ±SE (months)	95% CI (months)	*p*-value
VEGF-A	low-grade	118±9	89-154	0.005	107±8	94-133	0.044
	high-grade	78±7	41-98		58±9	48-108	
VEGF-B	low-grade	114±8	78-148	0.002	100±9	68-132	0.044
	high-grade	58±9	34-96		70±8	40-92	
VEGF-C	low-grade	115±6	78-154	0.006	100±8	72-133	0.035
	high-grade	76±8	34-99		70±6	32-92	
VEGF-D	low-grade	110±9	72-154	0.038	99±9	82-133	0.032
	high-grade	72±8	56-96		74±7	40-90	
PlGF	low-grade	113±7	89-154	0.026	100±8	78-133	0.019
	high-grade	78±7	42-99		68±7	52-98	
VEGFR-1	low-grade	108±7	76-154	0.018	98±9	68-133	0.008
	high-grade	70±9	32-96		64±8	21-89	
VEGFR-2	low-grade	109±8	78-154	0.017	90±6	72-133	0.041
	high-grade	70±9	36-96		70±8	31-96	
VEGFR-3	low-grade	115±7	72-154	0.032	88±10	74-133	0.047
	high-grade	78±8	36-96		66±7	34-108	

Notes: CRC, colorectal cancer; VEGF, vascular endothelial growth factor; VEGFR, VEGF receptor; IHC, immunohistochemistry; ELISA, enzyme-linked immunosorbent assay; low-grade, < median value of protein expression level in tumor tissue or serum; high-grade, > median value of protein expression level in tumor tissue or serum; CI, confidence interval.

Supplementary Table S4. VEGF and VEGFR antibodies for immunohistochemistry of CRC tissues

Antibodies	Catalog#	Host	Antigen	Dilution	Source
Anti-VEGFA	ab1316	Mouse	VEGFA	1:200	Abcam Plc, Cambridge, UK
Anti-VEGFB	ab135780	Rabbit	VEGFB	1:100	Abcam Plc, Cambridge, UK
Anti-VEGFC	ab9546	Rabbit	VEGFC	1:200	Abcam Plc, Cambridge, UK
Anti-VEGFD	ab63068	Rabbit	VEGFD	1:50	Abcam Plc, Cambridge, UK
Anti-PlGF	ab97618	Rabbit	PlGF	1:250	Abcam Plc, Cambridge, UK
Anti-VEGFR-1	ab32152	Rabbit	VEGFR-1	1:100	Abcam Plc, Cambridge, UK
Anti-VEGFR-2	ab39638	Rabbit	VEGFR-2	1:100	Abcam Plc, Cambridge, UK
Anti-VEGFR-3	ab72240	Mouse	VEGFR-3	1:200	Abcam Plc, Cambridge, UK

Notes: VEGF, vascular endothelial growth factor; VEGFR, VEGF receptor; CRC, colorectal cancer; PlGF, placental growth factor.

Supplementary Table S5. ELISA kits for analysis of serum VEGF, sVEGFR, and CEA proteins in the CRC patients

ELISA	Catalog#	Antigen	Source
Human VEGFA	ab119566	VEGFA	Abcam Plc, Cambridge, UK
Human VEGFB	KA3119	VEGFB	Abnova Co., Walnut, CA, USA
Human VEGFC	ab100664	VEGFC	Abcam Plc, Cambridge, UK
Human VEGF-D	ELH-VEGFD	VEGFD	RayBiotech, Inc. Norcross, GA, USA
Human PlGF	ab100629	PlGF	Abcam Plc, Cambridge, UK
Human VEGFR-1	ab119567	VEGFR-1	Abcam Plc, Cambridge, UK
Human VEGFR-2	ab100665	VEGFR-2	Abcam Plc, Cambridge, UK
Human VEGFR-3	ab100666	VEGFR-3	Abcam Plc, Cambridge, UK
Elecsys® CEA	11731629322	CEA	Roche Diagnostics, Switzerland

Note: ELISA, enzyme-linked immunosorbent assay; VEGF, vascular endothelial growth factor; VEGFR, VEGF receptor; sVEGFR, soluble VEGFR; CEA, carcinoma embryonic antigen (modular analytics, cobas e601); CRC, colorectal cancer; PlGF, placental growth factor.

Supplementary Figure S1. Changes in the levels of circulating vascular endothelial growth factors (VEGFs), placental growth factor (PlGF), and soluble VEGF receptors (sVEGFRs) were found in the postoperative colorectal cancer (CRC) patients who were treated with 12 cycles (2-week interval per cycle) of bevacizumab (5 mg/kg) plus FOLFIRI (5-FU 400 mg/m^2/day for 2 days, leucovorin 400 mg/m^2 for 1 day, and irinotecan 180 mg/m^2 for 1 day). Data were expressed as means±SD (n=5) after ELISA analysis. *, $P < 0.05$, *vs* cycle 1 at given VEGF or sVEGFR.

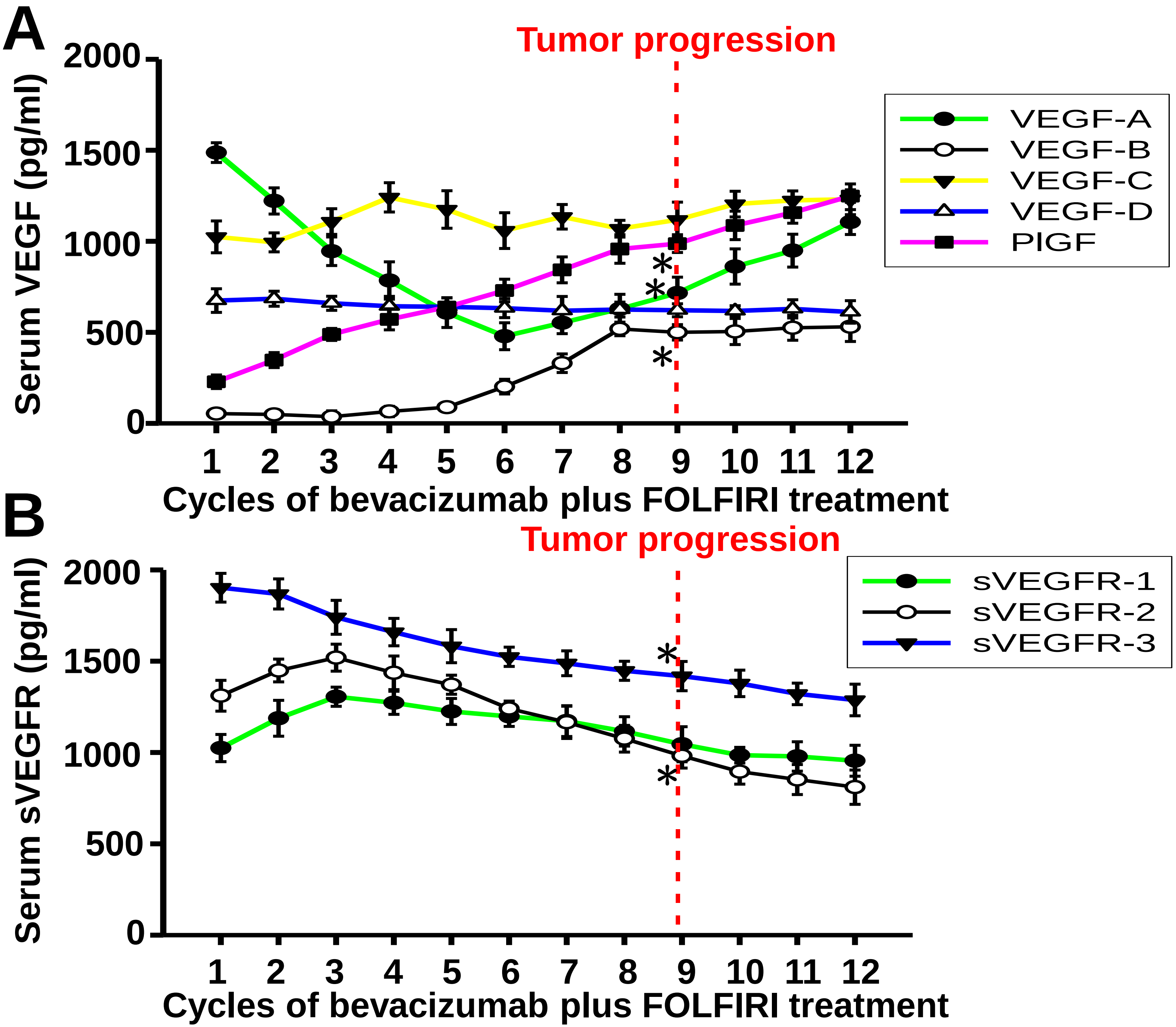
A
Tumor progression
Serum VEGF (pg/ml)
2000
1500
1000
500
0
1 2 3 4 5 6 7 8 9 10 11 12
Cycles of bevacizumab plus FOLFIRI treatment
VEGF-A
VEGF-B
VEGF-C
VEGF-D
PIGF
*
*
*
B
Tumor progression
Serum sVEGFR (pg/ml)
2000
1500
1000
500
0
1 2 3 4 5 6 7 8 9 10 11 12
Cycles of bevacizumab plus FOLFIRI treatment
sVEGFR-1
sVEGFR-2
sVEGFR-3
*
*
